# Direct radiocarbon dating and genetic analyses on the purported Neanderthal mandible from the Monti Lessini (Italy)

**DOI:** 10.1038/srep29144

**Published:** 2016-07-08

**Authors:** Sahra Talamo, Mateja Hajdinjak, Marcello A. Mannino, Leone Fasani, Frido Welker, Fabio Martini, Francesca Romagnoli, Roberto Zorzin, Matthias Meyer, Jean-Jacques Hublin

**Affiliations:** 1 Department of Human Evolution, Max Planck Institute for Evolutionary Anthropology, Deutscher Platz 6, D-04103 Leipzig, Germany; 2 Department of Evolutionary Genetics, Max Planck Institute for Evolutionary Anthropology, Deutscher Platz 6, D-04103 Leipzig, Germany; 3 Department of Archaeology, School of Culture and Society, Aarhus University, Moesgård Allé 20, 8270 Højbjerg, Denmark; 4 Università degli Studi di Milano-Bicocca, Piazza dell’Ateneo Nuovo 1, 20126 Milano, Italy; 5 BioArCh, University of York, York, YO10 5DD, United Kingdom; 6 Università degli Studi di Firenze, Dipartimento di Storia, Archeologia, Geografia, Arte e Spettacolo. Cattedra di Paletnologia (Prehistory), Via S. Egidio 21, 50122 Firenze, Italy; 7 Institut Català de Paleoecologia Humana i Evolució Social (IPHES) Zona Educacional 4–Campus Sescelades URV (Edifici W3) 43007 Tarragona, Spain; 8 Area de Prehistoria, Universitat Rovira i Virgili (URV), Avinguda de Catalunya 35, 43002 Tarragona, Spain; 9 Museo Civico di Storia Naturale di Verona, Sezione di Geologia e Paleontologia, Lungadige Porta Vittoria 9, 37129 Verona, Italy

## Abstract

Anatomically modern humans replaced Neanderthals in Europe around 40,000 years ago. The demise of the Neanderthals and the nature of the possible relationship with anatomically modern humans has captured our imagination and stimulated research for more than a century now. Recent chronological studies suggest a possible overlap between Neanderthals and anatomically modern humans of more than 5,000 years. Analyses of ancient genome sequences from both groups have shown that they interbred multiple times, including in Europe. A potential place of interbreeding is the notable Palaeolithic site of Riparo Mezzena in Northern Italy. In order to improve our understanding of prehistoric occupation at Mezzena, we analysed the human mandible and several cranial fragments from the site using radiocarbon dating, ancient DNA, ZooMS and isotope analyses. We also performed a more detailed investigation of the lithic assemblage of layer I. Surprisingly we found that the Riparo Mezzena mandible is not from a Neanderthal but belonged to an anatomically modern human. Furthermore, we found no evidence for the presence of Neanderthal remains among 11 of the 13 cranial and post-cranial fragments re-investigated in this study.

The process of replacement of Neanderthals by anatomically modern humans around 40,000 years ago in Western Eurasia is one of the most disputed topics in the field of Palaeoanthropology. Although the chronological overlap between the two groups likely lasted more than 5,000 years^1^,^2^, there is little evidence, if any, of a local coexistence for a significant amount of time. Nonetheless, careful evaluation of geographical scale and of the duration of local interactions between the two populations is critical to assess the possibility of biological admixture and cultural diffusion between ‘newly-arrived’ moderns and local archaics. As about 2% of Neanderthal ancestry is detected in the genomes of all present-day human populations outside of Africa, the Levant is considered one of the most likely areas where gene flow between Neanderthals and anatomically modern humans could have taken place^3^. Additional and regionally confined evidence of hybridization comes from a 37,000–42,000 years old modern human from Peştera cu Oase (Romania), which was shown to have had a Neanderthal ancestor four to six generations earlier based on the analysis of its genome^4^. Similar cases of local hybridization have been alleged in various parts of Europe based on anatomical or archaeological evidence, but they have not yet been substantiated by palaeogenetic data^5^,^6^. One example is the late Mousterian site of Riparo Mezzena in northern Italy, where archaeological and radiometric studies suggest local coexistence of anatomically modern humans and late Neanderthals (see ref. 7 for a summary). These studies were later bolstered by the morphological and palaeogenetic analysis of a fragmentary mandible recovered at the site (IGVR 203334), which was hypothesized to represent the remains of a Neanderthal whose ancestors had interbred with anatomically modern humans^8^.

Riparo Mezzena is a rockshelter located at ca. 200 m above sea level in the Monti Lessini mountain range, about 8 km from Verona in northern Italy (Fig. 1). The archaeological site was discovered in 1957 by Prof. Franco Mezzena, hence the site name^9^, and is one among several important Palaeolithic sites in the Monti Lessini territory, which also include Riparo Tagliente, Grotta della Ghiacciaia and Grotta di Fumane^10^,^11^,^12^. The archaeological stratigraphic sequence of Riparo Mezzena is composed of three layers^9^,^13^,^14^. The lowermost and middle layers, layers III and II respectively, contain diagnostic Mousterian lithic industries. These levels are overlain by layer I, which was found to contain a mixture of Palaeolithic and later pre- and proto-historic artefacts, including ceramics attributable to the Iron Age. During the study of the faunal assemblage by Angelo Pasa, nine bone fragments from layer I and four fragments from the other two layers were identified as human. These 13 remains include an incomplete mandible, 11 cranial fragments and one post-cranial bone fragment. A monograph by Corrain^15^ tentatively attributed the fragmentary mandible IGVR 203334 to a female Neanderthal, presumably based on the presence of Mousterian lithic industries in all three layers rather than on the size and morphology of the specimen, which displays possibly modern features (Fig. 2)^15^.

During the last ten years, Monti Lessini human remains have been re-examined by means of anatomical and palaeogenetic analyses^8^,^16^,^17^,^18^,^19^,^20^. This work was complemented by a new study of the lithic assemblage^21^. Based on the amplification of the hypervariable region 1 of the mitochondrial genome by PCR^16^, one of the cranial fragments, MLS 1, was shown to carry Neanderthal-like mitochondrial DNA (mtDNA). The mitochondrial sequence of MLS 1 was reproduced in later studies, accompanied by the successful retrieval of short stretches of nuclear gene sequences from the same specimen^18^,^20^. In addition, retrieval of Neanderthal mitochondrial and nuclear DNA was also reported for MLS 3^19^, and a short Neanderthal mtDNA sequence was obtained from the mandible^8^. Together these reports support the notion that the Monti Lessini material belongs to one Neanderthal individual. However, the evidence regarding the age of the material is based only on a single radiocarbon date (RTT-5578: ^14^C Age 34,540 ± 655; (68.2%) 39,870-38,420 calibrated years before present (cal BP); (95.4%) 40,780-37,480 cal BP) obtained on a bovid bone from layer III^7^. Unfortunately this faunal sample was not directly associated with the human remains of layer I, but came from the lowermost part of the Mezzena sequence. Nonetheless, its relatively young age in the broader context of the European Mousterian was interpreted as evidence for the presence of late-surviving Neanderthals in the Monti Lessini area^7^, possibly contemporaneous with anatomically modern human makers of Proto-Aurignacian industries from the neighbouring site of Fumane, dated between ca. 41,000 to 38,000 cal BP^22^. This, together with the genetic characterization of the human remains as Neanderthal, and the ambiguous anatomical features, led Condemi and collaborators^8^ to hypothesize interbreeding between Neanderthals and anatomically modern humans in this part of northern Italy.

The initial objective of our research was to provide a direct radiocarbon date on the human bone fragments from Riparo Mezzena, to better establish the chronology of the site. The unexpected results of this radiocarbon study urged us to undertake additional analyses, including ancient protein analysis with ZooMS (“Zooarchaeology by Mass Spectrometry”), extraction of ancient DNA and a re-assessment of the lithic assemblage from layer I, from which the human remains originated. The results of this interdisciplinary work are presented in this paper.

## Results

For this study, 10 of the 13 human specimens retrieved by Angelo Pasa were made available to us by the Natural History Museum of Verona. In addition, we were able to analyse a second post-cranial human fragment (IGVR 63017-5/MLS 3)^19^ belonging to the Riparo Mezzena collection, which was not described in Corrain^15^ and was probably identified during a recent re-examination of the faunal assemblage of Riparo Mezzena^23^. The two bone fragments that were not analysed in this study, which include MLS 1 (IGVR 63017-7 or IGVR 63017-9)^7^,^24^, used in most palaeogenetic studies, had not been returned to the above-mentioned Museum.

### ^14^C and stable isotope analysis

An important issue to consider when we approach an archaeological site with radiocarbon is the stratigraphic information we have at our disposal. For this reason it is important to remember that layer I, the uppermost part of the deposit from where almost all the human remains originate, was initially interpreted as partly formed by a pedological bioturbation of the underlying layer II. Layer I was additionally affected by anthropic disturbances, notably during proto-historic times, and Iron Age ceramics have been mentioned as originating from this layer alongside Palaeolithic artifacts^9^,^13^. During the excavation, the layer was not sub-divided into an upper and a lower part as proposed by Longo and collaborators^7^, who assigned the human remains to a purported sub-layer I b (L. Fasani personal communication; and ref. 13). Almost all of the human remains analysed in this study belong to layer I (see Table 1 and Supplementary Fig. S1).

A direct radiocarbon dating was attempted on four cranial fragments and on the mandible IGVR 203334. Three of the five samples selected for AMS radiocarbon dating have elemental concentrations (%C and %N) and C:N ratios (Table 1) that fall within the accepted ranges for well-preserved collagen based on the quality criteria proposed by van Klinken^25^ (see methodological section below). This was not the case for mandible IGVR 203334 and the skull fragment IGVR 63017-12. In these two specimens the yields of collagen were acceptable (1.3% for the mandible and 0.8% for the skull), but the C:N ratio was too high (4.1 and 3.7 respectively). However, given the importance of these specimens we decided to submit them for radiocarbon dating (see Supplementary Information for a more detailed discussion).

As a whole, the radiocarbon dates of the three successful samples are surprisingly young for a Mousterian site, with two samples dating to around 5,500 and one to 25,530 ± 107 ^14^C BP (Table 1). Moreover, the IGVR 203334 mandible with a non-acceptable C:N ratio, resulted in an age of 5,580 ± 26 ^14^C BP, which overlaps in 1σ with the two cranial fragments (IGVR63017-15 and IGVR63017-2) around 5,500 ^14^C BP, passing the isotopic criteria mentioned above. These three dates fall within the Neolithic time period. The other cranial fragments (IGVR 63017-12) with poorly preserved collagen and a C:N ratio just slightly above the acceptable ranges (Table 1), produced an age of 10,190 ± 33 ^14^C BP. These results suggest that the directly dated human bone fragments (including the mandible IGVR 203334, see Supplementary Information) are unlikely to belong to a Neanderthal, given that this group became extinct long before the Holocene.

We also performed stable isotope analyses on the human bones sampled for radiocarbon dating (see Supplementary Information for details). Carbon and nitrogen isotope data from well-preserved collagen, as proposed by van Klinken^25^, is only available from the parietal (IGVR 63017-15/S-EVA 32613), the occipital (IGVR 63017-2/S-EVA 32614) and the temporal (IGVR 63017-4/S-EVA 32615) bone fragments (Table 1). The first two specimens have identical isotopic compositions, which may indicate that they belonged to the same individual. Their δ^15^N values fall well within the known isotopic range of Neolithic individuals from the Italian Peninsula (e.g.^26^,^27^,^28^). For IGVR 63017-4 the nitrogen isotope value is low compared not only to the values of European Neanderthals and Upper Palaeolithic humans^29^,^30^,^31^, but also to those of Neolithic humans from the Italian Peninsula^26^,^27^,^28^. In fact, a δ^15^N value of 5.7‰ is a more likely isotope composition for an Upper Palaeolithic herbivorous or omnivorous animal than for a human from that period.

The doubts raised by the radiocarbon dating and isotope analyses on IGVR 63017-4 were among the reasons that pushed us to undertake ZooMS and DNA analyses on the purported Neanderthal bone fragments from Riparo Mezzena.

### ZooMS

Of the eight bone specimens tested, seven resulted in complete or partial peptide marker series (Table 2). Specimen IGVR 63017-5-MLS 3 returned an empty MALDI-TOF-MS spectrum. Visual inspection of this specimen raised the possibility that it might have been burned, but further analyses would be required to test this hypothesis. Among the other seven specimens, three were identified as hominin (IGVR 63017-3, IGVR 63017-11 and IGVR 63017-14), one as a Suidae (IGVR 63017-4), one as an ungulate (Cervid/Saiga^32^) (IGVR 63017-12), and two as Carnivora (Mustelidae/Pantherinae/Hyaenidae^32^) (IGVR 63017-1 and IGVR 63017-8). The latter identification could not be narrowed down further due to the absence of peptide marker A for both spectra. The Cervid/Saiga concerns a “complete” peptide marker series for the genera *Cervus*, *Megaloceros*, *Alces*, *Dama* and *Saiga*, but this cannot be resolved further taxonomically^32^. An attribution to *Cervus elaphus* seems probable based on taxonomic identifications of the faunal remains from the site^13^. Similarly, various carnivores are present at the site (*Canis lupus*, *Felis silvestris*, *Crocuta* sp.), which could correspond to the two carnivore bone specimens identified here (IGVR 63017-1 and IGVR 63017-8). The hominin identifications were supported by the presence of peptide marker A (1235.6 m/z), B (1477.8 m/z), D (2115.1 m/z), E (2832.4 m/z) and G (2957.5 m/z). In these spectra, we did not observe the presence of non-hominin markers.

### Ancient DNA

DNA was extracted from nine Mezzena specimens, which included the five fragments that were directly radiocarbon dated and four fragments identified by ZooMS (Table 2). Between 9.6 mg and 21.6 mg of bone powder were removed from the specimens for DNA extraction^33^. DNA libraries were generated using a highly sensitive single-stranded library preparation method^34^, enriched for human mitochondrial DNA (mtDNA)^35^ and sequenced on Illumina’s MiSeq or HiSeq platforms. Full-length molecule sequences were reconstructed from overlapping paired-end reads and aligned to the revised Cambridge reference sequence of the human mtDNA genome (rCRS, NC_0120920) using BWA^36^. In total, we obtained between 2,774 and 7,903 unique mitochondrial sequences from each of the specimens (Supplementary Table S1). To determine whether some of these sequences are of ancient origin, we next established the frequency at which cytosines (C) in the reference genome are substituted by thymines (T) in each position of the aligned sequences. Elevated frequencies of C to T substitutions are expected to occur in genuine ancient DNA due to deamination of cytosines to uracils (U), particularly in single-stranded overhangs at the ends of DNA fragments^37^, and are largely absent in recent contamination^38^,^39^. Substantial signals of cytosine deamination were observed in four of the Mezzena specimens: IGVR 20334 (the mandible), IGVR 63017-15, IGVR 63017-3 and IGVR 63017-14 (Supplementary Table S1). These frequencies increase substantially when filtering for sequences that have a C to T substitution at the opposing end (Supplementary Table 1), an observation that is consistent with the presence of both endogenous ancient DNA as well as present-day human contamination in the specimens^40^,^41^. We did not find any evidence for ancient human DNA preservation in the remaining five specimens, including those identified as Suidae and Cervid/Saiga by ZooMS.

Since too few putatively deaminated sequences are available to reconstruct complete mitochondrial genomes, we focused our analyses on sequences overlapping phylogenetically informative sites in the mtDNA genome. In a first branch-specific analysis, we looked at sites where the mtDNA genomes of modern humans, Neanderthals, and the Denisovan/Sima de los Huesos (SH) clade share derived variants that set them apart from all of the other hominin groups and the chimpanzee^41^. All of the sequences that overlap positions that are derived only in Neanderthals support the ancestral, i.e. non-Neanderthal state (Fig. 3). Likewise, there are no sequences supporting the Denisovan/SH state. We detect, however, strong support (100%) for the modern human-specific state in the sequences from all four specimens.

The support for the modern human lineage persists when taking into account only sequences showing evidence of deamination, *i.e.* after enriching for endogenous ancient DNA, but for two of the specimens (IGVR 63017-3 and IGVR 63017-15) this filter leaves only one informative sequence. In order to increase the resolution of the analysis, we expanded the set of diagnostic sites to those that differentiate between Neanderthal and modern human mtDNA only^42^, thereby assuming that the Mezzena specimens belong to one of the two lineages and not another group of hominins. When restricting to putatively deaminated sequences, all specimens again fully support the modern human state (Supplementary Table S2). Based on these results we conclude that IGVR 20334 (the mandible), IGVR 63017-15, IGVR 63017-3 and IGVR 63017-14 carry authentic ancient mtDNA of the modern human type.

### Lithic assemblage of layer I: A revision

The re-examination of the lithic assemblage from layer I, conducted as part of the present study to determine if traces of occupation from different prehistoric periods can be detected in the material culture (see Supplementary Information), clearly demonstrates the presence of diagnostic elements attributable to Holocene assemblages alongside Mousterian industries (Supplementary Figs S2 and S3). No detailed information is available for both the horizontal and vertical spatial distribution of these artefacts and further analyses are needed to interpret the site formation processes. Nevertheless, the evidence at hand suggests that the doubts cast on the integrity of layer I in the original publication on the site^9^ and in a second more detailed work^13^ were justified.

## Discussion

In this study we made use of a combination of state-of-the-art scientific methods (i.e. ^14^C dating, ZooMS, ancient DNA, and isotope analyses), to correctly assign the human remains from Riparo Mezzena both chronologically and taxonomically. We first note that the radiocarbon dates obtained from two of the bones that we sampled at the Natural History Museum of Verona show that they are much younger than expected for Neanderthal remains. The isotopic values on the same specimens suggest that they may belong to a single individual of Neolithic age. Furthermore, four of the 11 bones were identified as animals based on ZooMS, and four others (including mandible IGVR 203334) belong to anatomically modern humans and not Neanderthals according to the analysis of their DNA (Table 2).

**Figure 2:**
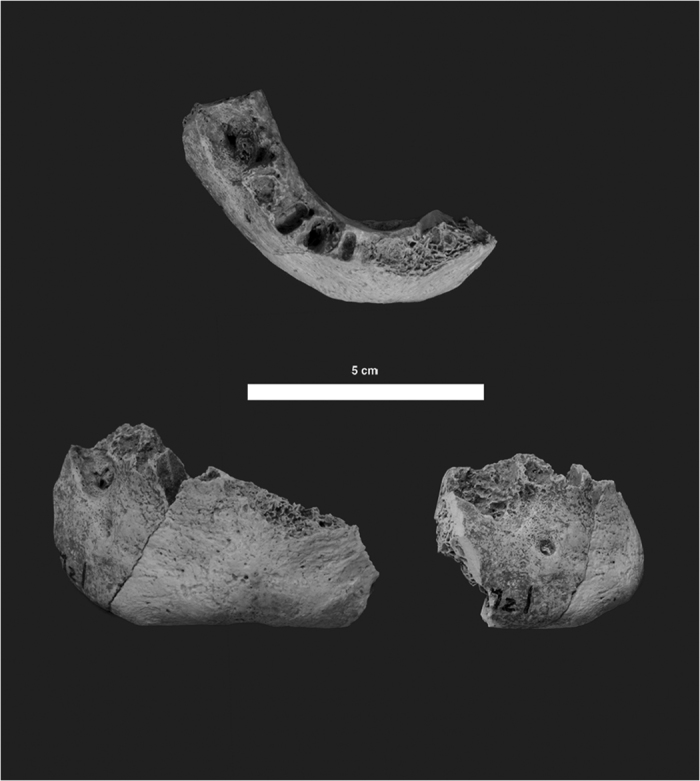
Riparo Mezzena mandible. IGVR 203334 specimen, top centre: superior view; bottom left: frontal view; bottom right: lateral view. The photos of the mandible were authorized by the Ministry for Cultural Heritage and Activities-Soprintendenza for Archaeological Heritage of Veneto, and taken by J-J. Hublin; reproduction forbidden.

**Figure 3:**
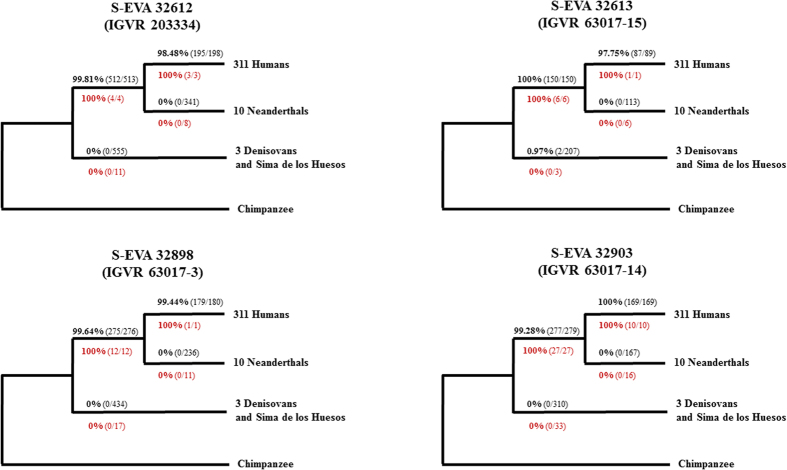
Assessment of the phylogenetic position of the Mezzena specimens IGVR 20334, IGVR 63017-15, IGVR 63017-3 and IGVR 63017-14 in the hominin mitochondrial tree based on phylogenetically informative (‘diagnostic’) positions. Reported are the percentages of sequences that support the derived state at positions that differentiate each of the branches from all others. The number of sequences supporting the derived state and the total number of sequences are denoted in brackets. Black numbers above the branches were determined using all mtDNA sequences, whereas red numbers below the branch are based only on sequences with terminal C to T substitutions.

Considering the morphological variability of the mandible in both modern humans and Neanderthals and the fragmentary nature of the mandible IGVR 203334, the new palaeogenetic results are fully compatible with the morphology of the Mezzena mandible (Fig. 2). The original anthropological study of the human mandible from Riparo Mezzena^15^ already indicated its modern features. Condemi and collaborators^8^ pointed out that some measurements taken on the specimen are within the variation of European Neanderthals. However, these measurements also fall within the variation observed in modern humans. Furthermore, the robusticity index at the mental foramen of IGVR 203334 displays a modern value outside of the Neanderthal range provided by Condemi and collaborators^8^. In the chin area, the mandible also displays a clear *trigonum mentale* of which the protrusion is undocumented in Neanderthals. Finally, and more importantly, the geometric morphometric analysis of the specimen conducted by A. Mounier in Condemi and collaborators^8^ established a primarily modern shape of IGVR 203334. The discriminant function analysis (DFA) classifies the specimen as anatomically modern human (Table S7 of Condemi *et al.*^8^), and in the Fig. 2 of Condemi *et al.*^8^ the Mezzena mandible is positioned outside of the cloud of Neanderthals and well within the cloud of anatomically modern humans.

The discrepancies between the results of the genetic analyses performed here and in previous studies^8^,^19^ are striking. The fact that we did not detect authentic ancient DNA in MLS 3 using the most sensitive method currently available^33^ is difficult to reconcile with the presence of ancient DNA in the specimen. Moreover, the mandible, which exhibits poor but detectable levels of ancient DNA preservation in our analysis, carries mtDNA of the modern human type. It is important to note that previous work relied on amplification of short stretches of DNA by PCR, an approach that is much less sensitive than current library preparation and high-throughput sequencing techniques^33^. Unlike PCR, library preparation allows molecules to be sequenced in their entirety, thereby providing information on DNA degradation patterns that lend evidence to the ancient origin of the modern human sequences retrieved from the Mezzena mandible. Our results highlight once more that PCR-based ancient DNA analyses are prone to contamination^43^. Yet, the case of Mezzena is unusual in that contamination must have been repeatedly introduced through PCR products of Neanderthal DNA rather than genomic DNA from modern humans. It is unfortunate that MLS 1, the specimen studied most extensively by means of genetics^16^,^18^,^20^, is not available for repeated analyses. The fact that the published mtDNA sequence of MLS 1 differs from the sequences of other Neanderthals^44^ does not *per se* prove its authenticity. The MLS 1 sequence was reconstructed from several short PCR products and it is conceivable that it represents a patchwork of contaminant Neanderthal and modern human sequences rather than a genuine Neanderthal sequence. Our findings thus put a question mark over all previous genetic results obtained from the Mezzena remains.

Based on the concordant results of the suite of techniques employed in our study, we do not support the hypothesis put forward by Condemi and collaborators^8^ that Riparo Mezzena and its surroundings was an area of long chronological overlap, where interbreeding between Neanderthals and anatomically modern humans took place. New excavations are required to gain a better understanding of crucial periods, such as the Middle-to-Upper Palaeolithic transition. If materials from sites excavated long ago (e.g. Riparo Mezzena) are to be used to provide additional data on these complex phases of our evolutionary history, then it should only be done using the whole suite of state-of-the-art methods at our disposal.

## Materials and Methods

At the Natural History Museum of Verona eight cranial fragments, two post-cranial samples and the human mandible from Riparo Mezzena were selected to perform the radiocarbon dating and the isotope analyses, the ZooMS and the ancient DNA analyses, described in the paper (Fig. 2 and Supplementary Fig. S1).

### Radiocarbon dating and isotope analysis

Five samples, including the mandible, were carefully pretreated, for the extraction of collagen and the subsequent isotopic study using the method described in Talamo and Richards^45^.

The outer surface of the bone samples are first cleaned by a shot blaster and then a 500 mg piece of bone is taken. The samples are then decalcified in 0.5 M HCl at room temperature until no CO_2_ effervescence is observed. 0.1 M NaOH is added for 30 minutes to remove humics. The NaOH step is followed by a final 0.5 M HCl step for 15 minutes. The resulting solid is gelatinized following Longin^46^ at pH3 in a heater block at 75 °C for 20 h. The gelatine is then filtered in an Eeze-Filter™ (Elkay Laboratory Products (UK) Ltd.) to remove small (>80 μm) particles. The gelatine is then ultrafiltered^47^,^48^ with Sartorius “VivaspinTurbo” 30 KDa ultrafilters. Prior to use, the filter is cleaned to remove carbon containing humectants^49^. The samples are lyophilized for 48 hours. In order to determine potential diagenetic alterations or contaminations of the collagen, the isotopic values (δ^13^C; δ^15^N), the elemental concentrations (%C; %N), the yield of collagen, and the C:N ratio must be evaluated^25^,^50^. Approximately 0.05 mg of the extracts were weighed out for stable isotope analysis, using a Thermo Finnigan Flash EA coupled to a Delta V isotope ratio mass spectrometer at the Max Planck Institute for Evolutionary Anthropology (MPI-EVA), Leipzig (Germany) (Lab Code: S-EVA). DeNiro^50^ showed that the C:N ratio should be between 2.9 to 3.6 for acceptable ranges, while van Klinken^25^ constrained this range to between 3.1 and 3.5. The yield of collagen that reflects the minimum level of well-preserved collagen, should start from 0.5% and go higher than 1%^25^. Here we consider collagen higher than 1% and C:N ratio between 2.9 and 3.6, as acceptable ranges for good quality collagen.

Between 3.0 and 6.0 mg of all extracts that meet the quality criteria described above were then weighed into pre-cleaned tin capsules and sent to the Klaus-Tschira-AMS facility of the Curt-Engelhorn Centre in Mannheim (Germany) (Lab Code: MAMS), where the subsequent graphitization step and AMS radiocarbon dating were undertaken^51^. All dates were corrected for residual preparation background estimated from pretreated ^14^C free bone samples, kindly provided by the MAMS and pretreated in the same way as the archaeological samples.

### DNA extraction and library preparation

Samples of between 9.6 mg and 21.6 mg of bone powder from nine Mezzena specimens were used for DNA extraction^33^ with modifications described in Korlević *et al.*^52^. Powder from one of the specimens, IGVR 203334, was treated with phosphate buffer^52^ prior to DNA extraction in order to eliminate some of the microbial DNA contamination from the sample. Twenty percent (10 μL) of each extract were used to prepare DNA libraries with a single-stranded DNA library preparation method^34^,^52^. The number of DNA molecules in each library was determined by digital droplet PCR (QX200 system, Bio-Rad), using 1 μL of 5,000-fold library dilution as template in an Eva Green assay (Bio-Rad, QX200 ddPCR EvaGreen Supermix) with primers IS7 and IS8^53^. All libraries were amplified and labelled with a pair of unique index sequences^54^ using AccuPrime Pfx DNA polymerase (Life Technologies)^55^ with the modifications described in Korlević *et al.*^52^. Half of the amplification products (50 μL) were purified using the MinElute PCR purification kit (Qiagen) and library concentrations were measured on the NanoDrop 1000 Spectrophotometer.

### ZooMS

Bone specimens (n = 8; IGVR 63017-4; IGVR 63017-12; IGVR 63017-3; IGVR 63017-11; IGVR 63017-14; IGVR 63017-1; IGVR 63017-8 and IGVR 63017-5) from Riparo Mezzena were analysed using ZooMS to test their taxonomic affiliation. Two of these samples were analysed because of their isotopic signature, which was inconsistent with the diets of the species to which they were attributed based on morphological identification (IGVR 63017-4 and IGVR 63017-12), while six additional specimens were analysed to screen for hominin specimens for subsequent genetic analysis. Between 2–20 mg of bone powder was demineralised in 0.5 M HCL and further processed following Welker *et al.*^32^. Taxonomic identifications were obtained through comparison of MALDI-TOF-MS spectra (900–4000 m/z) with peptide marker masses published previously^56^,^57^. A blank was processed alongside archaeological samples to monitor possible protein contamination during extraction.

## Additional Information

**Accession Code**: The sequences obtained in this study have been submitted to the European Nucleotide Archive (ENA SRA) under the accession number PRJEB14207.

**How to cite this article**: Talamo, S. *et al.* Direct radiocarbon dating and genetic analyses on the purported Neanderthal mandible from the Monti Lessini (Italy). *Sci. Rep.* **6**, 29144; doi: 10.1038/srep29144 (2016).

## Supplementary Material

Supplementary Information

## Figures and Tables

**Figure 1:**
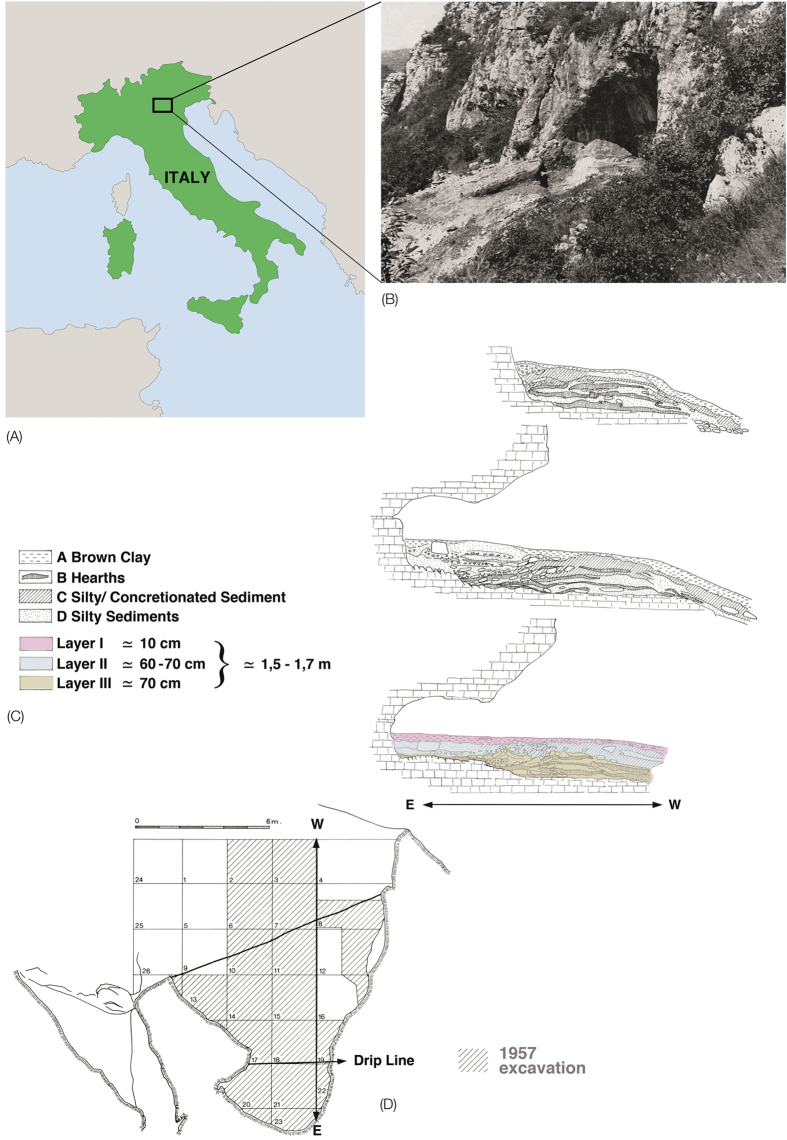
Location, view of entrance, stratigraphic sequence and the plan of Riparo Mezzena. (**A**) Location of the site in the Monti Lessini (northern Italy). (**B**) Photograph of Riparo Mezzena during the first excavation season in 1957 at the entrance of the Vajo Gallina. (**C**) Riparo Mezzena section drawing by A. Pasa in 1957. (**D**) 1957 Mezzena excavation area. The map of Italy is under MPI-EVA copyright. One original picture of the cave entrance (**B**) and the two modified pictures from Bartolomei *et al.*^13^ pictures (**C,D**) are authorized by the Natural History Museum of Verona; reproduction forbidden.

**Table 1:** Radiocarbon dates, isotopic values, % of collagen and C:N ratios of 5 purported human samples from Riparo Mezzena.

Museum reference Number	Anatomic Element	MPI Lab Code	Coll %	δ^13^C	δ^15^N	%C	%N	C:N	AMS Lab Code	^14^C Age	Err 1σ	68.2% cal BP from-to	95.4% cal BP from-to
IGVR 203334	Mandible	S-EVA 32612	1.3	−21.8	7.1	8.2	2.4	4.1	MAMS-24343	5,580	26	6,400-6,310	6,410-6,300
IGVR 63017-15	Left Parietal fragment	S-EVA 32613	2.9	−20.7	9.3	34.7	12.1	3.4	MAMS-24344	5,675	23	6,490-6,410	6,500-6,400
IGVR 63017-2	Occipital	S-EVA 32614	4.5	−20.7	9.3	38.2	13.3	3.3	MAMS-24345	5,530	23	6,390-6,290	6,400-6,280
IGVR 63017-4	Left Temporal fragment	S-EVA 32615	1.4	−20.4	5.7	30.2	10.0	3.5	MAMS-24346	25,530	107	29,800-29,440	30,090-29,290
IGVR 63017-12	Cranial fragment	S-EVA 32616	0.8	−21.2	6.6	17.8	5.6	3.7	MAMS-24347	10,190	33	11,980-11,810	12,050-11,750

Of these samples, only IGVR 203334, IGVR6 3017-15 and IGVR 63017-2 belong to modern humans, while the last two belong to Suidae and Cervid/Saiga (see Table 2). The isotopic values, C:N ratios and the amount of collagen extracted (Coll %) refer to the >30 kDa fraction. δ^13^C values are reported relative to the vPDB standard and δ^15^N values are reported relative to the AIR standard. The calibration was computed by OxCal 4.2^58^ using the International Calibration Curve IntCal13^59^.

**Table 2:** ZooMS and mtDNA identification on 11 of the purported human bone samples from the Riparo Mezzena collection.

Museum reference Number	Level	Anatomic Element	mtDNA analysis	ZooMS analysis
IGVR 203334	Layer I	Mandible	Modern Human	Not analysed
IGVR 63017-1	Layer I-q.6	Occipital fragment	Not analysed	Mustelidae/Pantherinae/Hyaenidae
IGVR 63017-2	Layer II	Occipital fragment	No evidence for ancient human DNA	Not analysed
IGVR 63017-3	No Layer	Occipital fragment	Modern Human	Homininae
IGVR 63017-4	No Layer-Clandestine excavation	Left Temporal fragment	No evidence for ancient human DNA	Suidae
IGVR 63017-5-MLS 3	Layer I-q.6	Clavicle fragment	No evidence for ancient human DNA	No Collagen
IGVR 63017-8-MLS 4	Layer I	Vertebral fragment	Not analysed	Mustelidae/Pantherinae/Hyaenidae
IGVR 63017-11	Layer I-inside	Parietal fragment	No evidence for ancient human DNA	Homininae
IGVR 63017-12	Layer III	Cranial fragment	No evidence for ancient human DNA	Cervid/Saiga
IGVR 63017-14	Layer I-q.3	Parietal fragment?	Modern Human	Homininae
IGVR 63017-15	Layer I-q.7	Left Parietal fragment	Modern Human	Not analysed

ZooMS identifications, where available are consistent with observations based on mtDNA analysis and stable isotope analysis (see Table 1 and Supplementary Information).
