# Supplementary material for: Direct radiocarbon dating and genetic analyses on the purported Neanderthal mandible from the Monti Lessini (Italy)
Source: Sci Rep. 2016 Jul 8;6:29144. doi: 10.1038/srep29144 (PMC4937366; doi:10.1038/srep29144)
Supplement: Supplementary Information [file srep29144-s1.pdf]

SUPPLEMENTARY INFORMATION

Direct radiocarbon dating and genetic analyses on the purported Neanderthal mandible from the Monti Lessini (Italy)

Sahra Talamo, Mateja Hajdinjak, Marcello A. Mannino, Leone Fasani, Frido Welker, Fabio Martini, Francesca Romagnoli, Roberto Zorzin, Matthias Meyer, Jean-Jacques Hublin

Mezzena Rockshelter excavation history

Sahra Talamo, Leone Fasani and Roberto Zorzin

Riparo Mezzena is located in the Monti Lessini, at ca. 200 m altitude on the left slope of the Vajo Gallina in northern Italy (Fig.1 (A) in the main text). The rockshelter opens in the Eocene limestone cliff of Nummulitis complanata, several kilometres north of Avesa Valley, just 8 km away from the famous Shakespearian city of Verona.

The site was initially explored at the end of the 1950s by Prof. Franco Mezzena, hence its name. The formal excavation started in 1957 and was extended for the following three years under the direction of A. Pasa, F. Zorzi and F. Mezzena, with support from the Natural History Museum of Verona.

The first complete stratigraphic sequence was the object of a preliminary publication in 1960 by F. Zorzi, who described three stratigraphic levels[1]. Two diagnostic Mousterian levels at the bottom (layer III in contact with the bedrock of the shelter, followed by layer II), overlain by the uppermost layer I. This layer contained numerous ceramic fragments attributable to the Iron Age, as well as tools of Palaeolithic age (Fig.1 (C), (D) in the main text). In 1962 P. Leonardi and A. Broglio, published a more detailed study of the lithic industry[2]. During the study of the faunal assemblage A. Pasa found several human remains originating from the uppermost layer I, which

were subsequently published in Corrain[3]. The paper by this researcher describes these remains (i.e. 11 pieces of cranial fragments, one mandible and one piece of rib) in great detail (Fig. 2 in the main text and Supplementary Fig.S1). The discovery of the incomplete mandible, which was compared to the Neanderthal of Circeo III, is particularly noteworthy. The presence of a marked chin and the relative gracility of the specimen pushed Corrain[3] to point out that the mandible had traits of modernity and was very different from Neanderthal males. However, the presence of small diagnostic Neanderthal features and the substantial Mousterian lithic assemblage, which implied the presence of Neanderthals, resulted in the conclusion that the mandible was closer to the female Neanderthal of "*La Naulette*".

In 1977, the rockshelter was re-explored by A. Sartorelli in collaboration with G. Bartolomei, A. Broglio, C. Peretto, L. Cattani, B. Sala, G. Balboni and M. Cremaschi. The goal of this second investigation at Riparo Mezzena was to continue the excavation of the trenches left open from 1957, to better sample the deposits and their contents so that sedimentological and palynological analyses could be undertaken. However, it was only possible to locate the lower part of the sequence (ca. 70 cm of thickness corresponding to the Pasa-Zorzi-Mezzena excavation), because despite a fence built by the Natural History Museum of Verona to protect the archaeological site, clandestine excavations had severely damaged the top of the deposit left unexcavated by Pasa. It was therefore not possible to investigate the two upper layers and the understanding of these levels had to be of necessity based on the schematic notes from the original excavation and on the analysis of a sedimentological sample collected by A. Pasa in 1957. In 1980 the first and only detailed report on the site was published[4]. Riparo Mezzena is located in a thermoclastic niche, at most 10 m wide (Fig. 1 in the main text). As mentioned above, three different stratigraphic layers were identified ranging in thickness between 1.5 and 1.7 m. The basal level of the deposits

within the shelter (layer III) is ca. 70 cm thick and overlays the bedrock. The presence of different hearths in succession suggests the repeated use of the site by humans during the accumulation of this layer. At the top of this level there is evidence of a strong thermoclastic weathering episode followed by a concretion that marks the start of layer II, immediately above. Pollen and faunal analyses suggest that layer III accumulated during a period of prevalently humid and temperate continental climatic conditions. The fauna includes red deer, roe deer, cattle and boar, as well as small mammals. Layer III contains exclusively Mousterian lithics, such as side scrapers and side scrapers with a thinned back, and double carinated points (limaces) are also present. The Levallois flaking technique is the most common technological method in this layer. The thousands of artefacts from layer III can be assigned typologically to a typical Mousterian, falling within Group I and II of the Bordes classification scheme[4,5].

Layer II, which is 60-70 cm thick, is constituted by loess interbedded with at least three concretion layers. This level is sedimentologically very different from the underlying deposit and contains noticeably fewer traces of human occupation.

The sedimentological analysis of layer II attests the alternation of rather dry and cold climatic phases (represented by the loess levels) with humid climatic episodes (represented by the concretion levels). The faunal assemblage is represented by the same groups of mammals as the layer below. In addition, the skeletal remains of the rodent *Marmota mamota* were very abundant. This burrowing animal is represented by numerous specimens in both the upper layers (II and I), indicating that bioturbation was a serious site disturbance process at Riparo Mezzena. In fact, almost complete skeletons of *M. marmota* have been recovered, suggesting that Riparo Mezzena may have been home to a colony of these rodents at different times after the Middle Palaeolithic.

Few important differences in the lithic industries between layer III and II have been detected. This could be due to the limitations of the excavation techniques, which may have merged materials from different horizons into two thick layers. It is important to remember that, at the time of the excavation, wet sieving was not performed. It should be pointed out, however, that the lithic industries from this layer suggest that there was a regression in the Levallois flaking technique at the time of its deposition.

Layer I was interpreted as a pedological alteration of layer II, caused by recent bioturbation and disturbance to the deposit by its proto-historic human occupants. This is corroborated by the study of the faunal assemblage which, besides the taxa present in the underlying layers, also includes domestic dogs and goats. A. Pasa noted that layer I is ca. 10 cm below the herbaceous cover on top of the sequence, and it is a humid dark-brown soil, containing artefacts and bones attributable to the Middle-Upper Palaeolithic[4]. The few Mousterian lithics in layer I attests a further regression in the industries from the uppermost layers compared to those from the layers below. It should also be added that pottery assignable to the Iron Age period was found together with yet unstudied Upper Palaeolithic tools. The nature of the finds from this layer made it difficult to draw any meaningful conclusions on the chronology of its deposition, although it was clear that this part of the sequence must have been severely disturbed.

The lithic assemblage from Riparo Mezzena can be classified as follows according to the Bordes scheme[5]. Group I (Levallois group) is conspicuous in layer III, the typological Bordes index corresponds to 34 (Table (c) page 25 in Bartolomei et al.[4]) and is reduced in layer II (typological Bordes index =20.96; Table (c) page 30 in Bartolomei et al.[4]). Group II (Mousterian group) is the most abundant in both lower layers. The Bordes typological index for layer III is 72 and for layer II is 66.28; in this case too, there was thus a slight reduction in the index. Group III (Upper

Palaeolithic group) is rare, albeit remaining constant through the layers in question (layer III typological Bordes index =4.72; layer II typological Bordes index =4.4). In contrast, in Group IV (Denticulate group) there is a slight increase from bottom to top (layer III typological Bordes index =13.43; layer II typological Bordes index =15).

Radiocarbon discussion

Sahra Talamo

The expected age for late Neanderthal samples should be close to the limit of the ^{14}C method and only a massive contamination by modern carbon could have skewed Mousterian dates to the point of producing Neolithic ages. To attain an age of 5,578±26 ^{14}C BP from a sample of around 35,000 ^{14}C BP, 50% of the total carbon should originate from modern carbon. If the source of modern carbon were animal glue added for conservation purposes, it would imply a complete saturation of the specimens with glue. This is not the case, as the visual check of the specimen can attest, in fact there is a limited amount of glue on one of the broken edges of the mandible and along the break where the two mandibles fragments are joined, but the bone itself is not saturated at all and we sampled the part where the glue was not applied. Moreover, the carbon concentration in the mandible sample is 8.2%, which is quite low when compared to the accepted values for bone collagen. This is not indicative of contamination by carbon from modern sources, but rather of the presence of inorganic substances in the extract[6]. Another aspect to consider is the collagen yield, which, in the case of the mandible, should display a much higher value than 1.3% of collagen, if we hypothesize that this sample may have been soaked in animal bone glue produced during the 1950s. This relatively low yield of collagen associated with low isotope values and out of the range C:N ratio, indicates collagen degradation rather than contamination from modern carbon[6].

The other two cranial samples IGVR 63017-15 and IGVR 63017-2 (MAMS-24344 ^{14}C Age 5,675±23 BP and MAMS-24345 ^{14}C Age 5,530±23 BP respectively), which range within the same time period as the mandible, contained very well-preserved collagen. Similarly, the sample that yielded the oldest radiocarbon result IGVR 63017-4 (MAMS-24346 ^{14}C Age 25,526±107 BP) was also well-preserved. For this reason we consider that all of these samples contain biogenic collagen and we have used them to make tentative palaeodietary inferences on the human specimens from Riparo Mezzena (see below).

If the IGVR 63017-12 result (MAMS-24347 ^{14}C Age 10,190±33 BP) obtained on the last fragment, purportedly Mousterian, was contaminated with glue as well, this contamination should be in the order of 27% of modern carbon mixed with 73% of original human carbon. This is more plausible than the estimate discussed above for the mandible IGVR 203334, but remains inconceivably high. However, the combination of the low yield of collagen and the slightly high C:N ratio even in this case is more compatible with a degradation of the original collagen than with a modern contamination as discussed in van Klinken[6].

Stable Isotope analysis

Marcello A. Mannino

Stable isotope analyses, to reconstruct past human diets[7], were conducted on the bone collagen of the human specimens sampled for radiocarbon dating (Table 1). Carbon ($\delta^{13}C$) and nitrogen ($\delta^{15}N$) isotope analyses are an established method and when applied on bone collagen are mainly useful to establish the ecosystem of origin and trophic level of the dietary protein consumed. According to the quality criteria proposed by van Klinken[6], of the five human bones sampled for radiocarbon dating, the parietal IGVR 63017-15 (S-EVA 32613), the occipital IGVR 63017-2 (S-EVA 32614) and the temporal IGVR 63017-4 (S-EVA 32615) bone fragments can be

considered well-preserved. The first two specimens have identical isotopic compositions, which may indicate that they belonged to the same individual. The $\delta^{13}C$ value (= -20.7‰) is typical of individuals living in terrestrial environments dominated by C_3 plants, such as those of Europe, and indicates that the diet of this human was based on terrestrial protein. The $\delta^{15}N$ value (= 9.3‰) is difficult to interpret in the absence of isotopic values from animals contemporary to the human in question. However a growing body of isotope data on bone collagen at our disposal, allow us to make inferences on the diet of the human from Riparo Mezzena. A $\delta^{15}N$ value of 9.3‰ falls at the lower end of the dataset available for Neanderthals, who isotopically are considered as highly carnivorous (e.g.[8,9]). No isotope values are available for Neanderthals from the Italian Peninsula and the only specimen with a similar isotope composition to the Mezzena specimen is the Feldhofer 1 ($\delta^{15}N$ = 9.0‰), whilst the other individual from the Neanderthal type site (i.e. Feldhofer 2) has a lower value ($\delta^{15}N$ = 7.9‰)[10]. However, all other Neanderthals have $\delta^{15}N$ values ≥10.3‰ that indicate high levels of animal protein consumption[8]. On the other hand, the $\delta^{15}N$ values for the two cranial fragments from Mezzena fall well within the known isotopic range of Neolithic individuals from the Italian Peninsula (e.g.[11-13]). Neolithic diets were dominated by terrestrial foods and characterized by an isotopically detectable proportion of animal protein consumption, within which was likely a balanced diet that included significant proportions of plant foods.

The Neolithic individual, to whom the two cranial bone fragments recovered from Riparo Mezzena belonged, probably had a similar diet. It should also be pointed out that (because of the doubts raised here on the genetic and chronological attribution to *Homo neanderthalensis* of the bones from Mezzena) the preliminary interpretations of the isotope analyses of the human

remains from this site[14] should no longer be considered valid for the debate on dietary change during the Middle-to-Upper Palaeolithic transition.

Carbon and nitrogen isotope data from well-preserved collagen is also available from specimen IGVR 63017-4 (S-EVA 32615 $\delta^{13}C$ = -20.4‰; $\delta^{15}N$ = 5.7‰). The carbon isotope value for this specimen is compatible with local terrestrial environments, as noted for the human whose composition is discussed above. However, the nitrogen isotope value of this specimen is low compared not only to the values of European Neanderthals and Upper Palaeolithic humans[8-10], but also to those of Neolithic humans from the Italian Peninsula[11-13]. In fact, a $\delta^{15}N$ value of 5.7‰ is a more likely isotope composition for an Upper Palaeolithic herbivorous or omnivorous animal than for a human from that period. The doubt raised by the isotopic values on IGVR 63017-4 was subsequently confirmed by ZooMS analysis (see main text).

DNA analysis

Mateja Hajdinjak and Matthias Meyer

Enrichment of the mitochondrial DNA (mtDNA) and sequencing

Amplified libraries were enriched for human mitochondrial DNA using a capture protocol described in Fu et al.[15] and a set of probes based on the revised Cambridge Reference Sequence (rCRS, NC_012920). Enriched libraries were sequenced on Illumina's MiSeq and HiSeq 2500 platforms using double index configuration (2 x 76 cycles)[16].

Base calling was done using Bustard (Illumina) for the libraries sequenced on the MiSeq (R5296, R5297, R5298, R5299, R5300, R5303, R5563, R5564, R5565, R5566, R5569 and R5570) and freeIbis[17] for the libraries sequenced on the HiSeq (A9245, A9250 and A9251). Overlapping paired-end reads were merged into single sequences[18]. Merged sequences were mapped to the rCRS using the Burrows-Wheeler Aligner (BWA)[19] with parameters adapted for ancient DNA

sequences ("-n 0.01 –o 2 –l 16500")[20]. Subsequent analyses were restricted solely to sequences whose index readings perfectly matched one of the expected index combinations. PCR duplicates were removed using bam-rmdup (https://github.com/udo-stenzel/biohazard) by calling a consensus from fragments with identical alignment start and end positions. Unmapped sequences and those shorter than 35bp were discarded in downstream analyses.

Phylogenetic analysis

We investigated the state of sequences that overlapped four sets of 'diagnostic' positions following the methodology described in Meyer et al.[21]. A diagnostic position was defined as a position where all individuals of a particular hominin group differ from all of the individuals that are not part of this group. To determine these positions we used the mtDNA genomes of 311 present-day humans[22], 10 Neandertals[22-26], three Denisovans[27-29], one Sima de los Huesos individual[30] and the chimpanzee[31]. Furthermore, in order to increase the resolution of the analysis, we studied the state of sequences overlapping the fifth set of 'diagnostic' positions where 10 Neanderthal mtDNA genomes differ from all 311 present-day humans. Each analysis was carried out twice, using only unique sequences that were 35bp or longer and sequences with a C to T difference to the reference genome at the first and/or last position in the alignment.

Revision of the lithic assemblages of layer I

Fabio Martini and Francesca Romagnoli

The lithic collection found in layer I during the 1957 field campaign and the associated archives examined for this work are stored in Natural History Museum depot in Verona. The assemblage is composed of 6742 pieces and 60% of them are unidentifiable, short fragments.

All the artefacts are made of fine grained chert, most likely collected locally in the Mesozoic limestone formations of Maiolica, Scaglia Variegata and Scaglia Rossa and in the Eocene

Limestone, all present between 5 to 15 km from the site and dominant in the Riparo Mezzena archaeological sequence[32-34]. The lithics are characterized by irregular rounded surfaces, patinas, frequent micro-fragmentations of the edges and natural removals, suggesting that complex taphonomic processes have affected the deposit. The lithic finds have lengths between 11 mm and 101 mm. During the fieldwork campaigns, the sediments were sieved through dry screening; although the excavation methods used at the end of 1950s were probably not as meticulous as modern-day ones; the presence of many chert fragments and flakes approximately 10 mm long implies that the collection can be considered representative of the original assemblage in the deposit.

The technological features of the artefacts[35] suggest the use of three main production strategies: recurrent centripetal, unipolar and multidirectional core exploitations. Several elements (stored in drawer 34 of the Museum depot) can be attributed to Holocene technology (Supplementary Fig.S2). Blade production was not significant in the assemblage, and a large proportion of the products are flakes with low laminar index (value < 2) and high morphological variability. Nevertheless, the presence of two fragmented blades with marginal retouching, two blades with truncations and ten unretouched blades with sub-parallel lateral edges, triangular or trapezoidal transversal section, and punctiform or linear butt is noteworthy. Furthermore, three bladelet cores are present in the assemblage. We have also identified a trapezoid microlith produced with the microburin blow technique and "*piquant-trièdre*", and a second trapezoid geometric microlith that is partially fragmented. A fragment of sickle element 44 mm long made on a large blade shows an invasive unilateral pressure retouch with pronounced lustrous on the ventral surface. The typological structure of the assemblage, which is poorly constituted, is characterized by

beaks. Sporadic end-scrapers, not microlithic backed tools with truncations, and a fragment of backed tool with opposite shoulder have also been identified.

Within the lithic assemblage recovered in layer I, there are few elements that could be attributed to a Mousterian complex (Supplementary Fig. S3); more specifically, three recurrent centripetal Levallois cores and two recurrent unidirectional Levallois cores, two "Quina" scrapers, and three Mousterian points with stepped scaled retouch ("demi-Quina" style). They are stored in drawers 34 and 35 of the Museum depot. Levallois knapping modalities are characteristic of the underlying layers II and III and have been described in detail[4,32,34]. Similarly the demi-Quina retouch has been described within the techno-complex of layer II and III, which is attributed to the La Ferrassie *facies* of Charentian Mousterian[4,34].

References

1 Zorzi, F. in *Verona e il suo territorio* **1**, 73-153 (Istituto per gli Studi Storici Veronesi, 1960).

2 Leonardi, P. & Broglio, A. *Le paléolithique de la Vénétie*. (Università degli studi di Ferrara, 1962).

3 Corrain, C. Resti scheletrici umani del Riparo Mezzena. *Memorie del Museo Civico di Storia Naturale di Verona* **16**, 97-101 (1968).

4 Bartolomei, G. *et al.* Il Riparo Mezzena:(stratigrafia del deposito, sedimenti, pollini, faune, industrie). *Museo civico di storia naturale di Verona* **2**, (1980).

5 Bordes, F. Mousterian Cultures in France. *Science* **134** (1961).

6 van Klinken, G. J. Bone Collagen Quality Indicators for Palaeodietary and Radiocarbon Measurements. *Journal of Archaeological Science* **26**, 687-695 (1999).

7 Lee-Thorp, J. A. On isotopes and old bones. *Archaeometry* **50**, 925-950 (2008).

8 Richards, M. P. & Trinkaus, E. Isotopic evidence for the diets of European Neanderthals and early modern humans. *PNAS* **106**, 16034-16039 (2009).

9 Bocherens, H. in *The evolution of Hominin diets*. 241-250 (Springer, 2009).

10 Richards, M. P. & Schmitz, R. W. Isotope evidence for the diet of the Neanderthal type specimen. *Antiquity* **82**, 553-559 (2008).

11 Le Bras-Goude, G. *et al.* Stratégies de subsistance et analyse culturelle de populations néolithiques de Ligurie: approche par l'étude isotopique ($\delta^{13}C$ et $\delta^{15}N$) des restes osseux. *Bulletins et mémoires de la Société d'Anthropologie de Paris*, 43-53 (2006).

12 Lelli, R. *et al.* Examining dietary variability of the earliest farmers of South-Eastern Italy. *American journal of physical anthropology* **149**, 380-390 (2012).

13 Tafuri, M. A. *et al.* in *Proceedings of the British Academy.* Vol. **198**, (2015).

14 Mannino, M.A. *et al.* Hard times for Neanderthals? An isotopic investigation on the diet of the Late Mousterian humans that populated northern Italy during the Middle-to-Upper Palaeolithic transition. *European Society for the Study of Human Evolution* (inaugural meeting, abstract) **63**, (2011).

15 Fu, Q. *et al.* DNA analysis of an early modern human from Tianyuan Cave, China. *Proceedings of the National Academy of Sciences* **110**, 2223-2227 (2013).

16 Kircher, M., Sawyer, S. & Meyer, M. Double indexing overcomes inaccuracies in multiplex sequencing on the Illumina platform. *Nucleic acids research*, gkr771 (2011).

17 Renaud, G., Kircher, M., Stenzel, U. & Kelso, J. freeIbis: an efficient basecaller with calibrated quality scores for Illumina sequencers. *Bioinformatics*, btt117 (2013).

18 Renaud, G., Stenzel, U. & Kelso, J. leeHom: adaptor trimming and merging for Illumina sequencing reads. *Nucleic acids research* **42**, e141-e141 (2014).

19 Li, H. & Durbin, R. Fast and accurate long-read alignment with Burrows–Wheeler transform. *Bioinformatics* **26**, 589-595 (2010).

20 Meyer, M. *et al.* A high-coverage genome sequence from an archaic Denisovan individual. *Science* **338**, 222-226 (2012).

21 Meyer, M. *et al.* Nuclear DNA sequences from the Middle Pleistocene Sima de los Huesos hominins. *Nature* **531**, 504-507 (2016).

22 Green, R. E. *et al.* A Draft Sequence of the Neandertal Genome. *Science* **328**, 710-722 (2010).

23 Briggs, A. W. *et al.* Targeted retrieval and analysis of five Neandertal mtDNA genomes. *Science* **325**, 318-321 (2009).

24 Gansauge, M.-T. & Meyer, M. Selective enrichment of damaged DNA molecules for ancient genome sequencing. *Genome research* **24**, 1543-1549 (2014).

25 Prüfer, K. *et al.* The complete genome sequence of a Neanderthal from the Altai Mountains. *Nature* **505**, 43-49 (2014).

26 Skoglund, P. *et al.* Separating endogenous ancient DNA from modern day contamination in a Siberian Neandertal. *PNAS* **111**, 2229-2234 (2014).

27 Krause, J. *et al.* The complete mitochondrial DNA genome of an unknown hominin from southern Siberia. *Nature* **464**, 894-897 (2010).

28 Reich, D. *et al.* Genetic history of an archaic hominin group from Denisova Cave in Siberia. *Nature* **468**, 1053-1060 (2010).

29 Sawyer, S. *et al.* Nuclear and mitochondrial DNA sequences from two Denisovan individuals. *PNAS* **112**, 15696-15700 (2015).

30 Meyer, M. *et al.* A mitochondrial genome sequence of a hominin from Sima de los Huesos. *Nature* **505**, 403-406 (2014).

31 Horai, S. *et al.* Man's place in Hominoidea revealed by mitochondrial DNA genealogy. *Journal of Molecular Evolution* **35**, 32-43 (1992).

32 Giunti, P. & Longo, L. Prime considerazioni sulla variabilità del sistema tecnico Levallois dello strato III di Riparo Mezzena (Monti Lessini, Italia settentrionale). *Rivista di scienze preistoriche* **58**, 5-27 (2008).

33 Longo, L. & Giunti, P. Settlement dynamics and raw material exploitation during Middle Palaeolithic in the Lessini Mountains (Verona, Veneto, Italy). *Middle Palaeolithic Human Activity and Paleoecology: New Discoveries and Ideas. Acta Universitatis Wratislaviensis, Studia Archeologiczne* **42**, 387e410 (2010).

34 Longo, L. *et al.* Did Neandertals and Anatomically Modern Humans coexist in Northern Italy during the late MIS 3? *Quaternary International* **259**, 102-112 (2012).

35 Inizan, M.-L., Reduron-Ballinger, M. & Roche, H. *Technology and Terminology of Knapped Stone: Followed by a Multilingual Vocabulary Arabic, English, French, German, Greek, Italian, Portuguese, Spanish*. Vol. **5** (Cercle de Recherches et d'Etudes Préhistoriques, 1999).

Supplementary Figures

Supplementary Figure S1 – Bone specimens sampled at the Natural History Museum of Verona.

The photos of the bone samples were authorized by the Ministry for Cultural Heritage and Activities - Soprintendenza for Archaeological Heritage of Veneto, and taken by S. Talamo; reproduction forbidden.

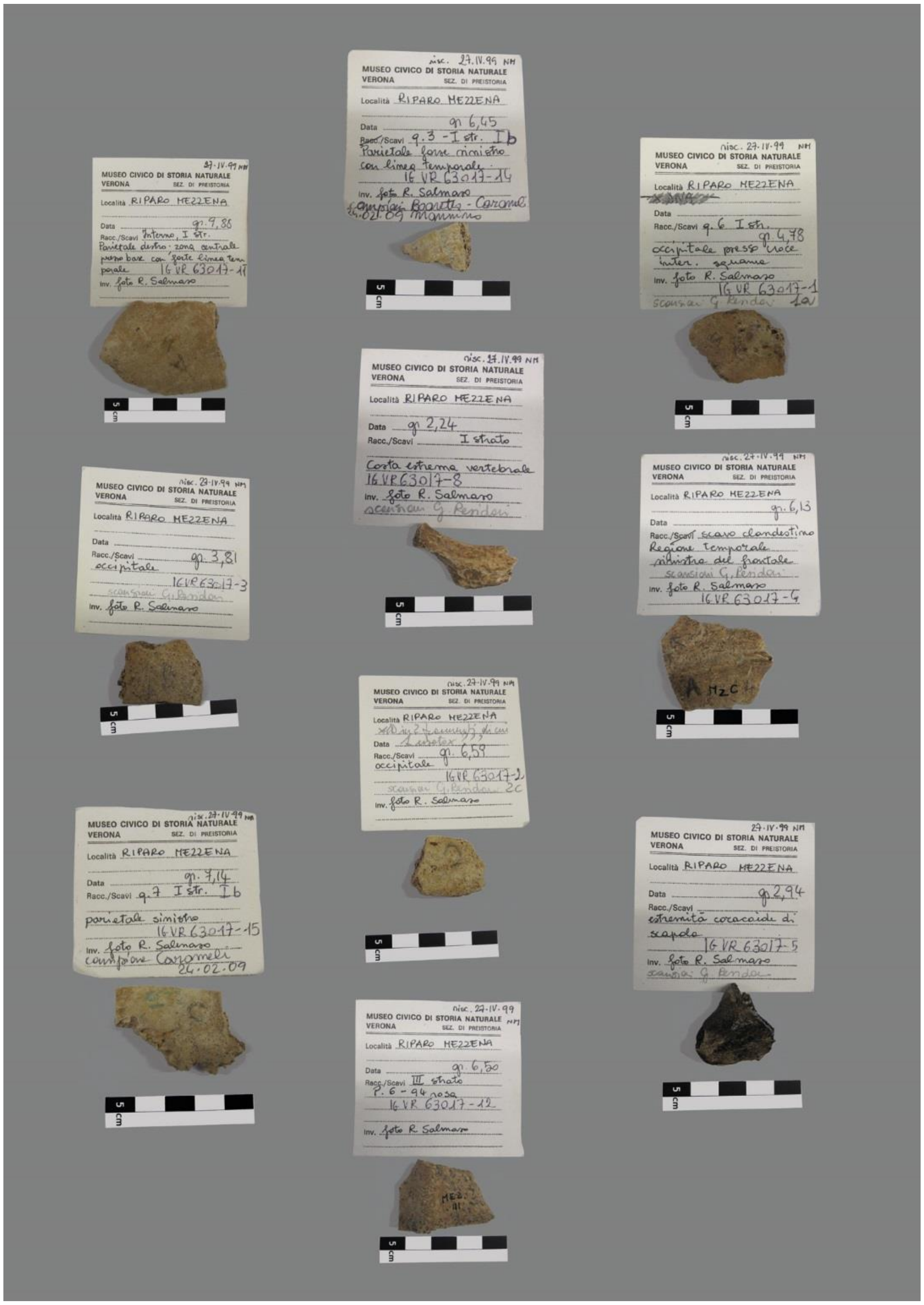

Supplementary Figure S2 – Riparo Mezzena, layer I. Holocene lithic remains. 1: Sickle element; 2: end-scraper; 3: trapezoid microlith with "*piquant-trièdre*"; 4: beak: 5-8: blades; 9: bladelet core. Scale bar 2 cm. The photos of the lithic assemblage were authorized by the Ministry for Cultural Heritage and Activities - Soprintendenza for Archaeological Heritage of Veneto, and taken by F. Romagnoli; reproduction forbidden.

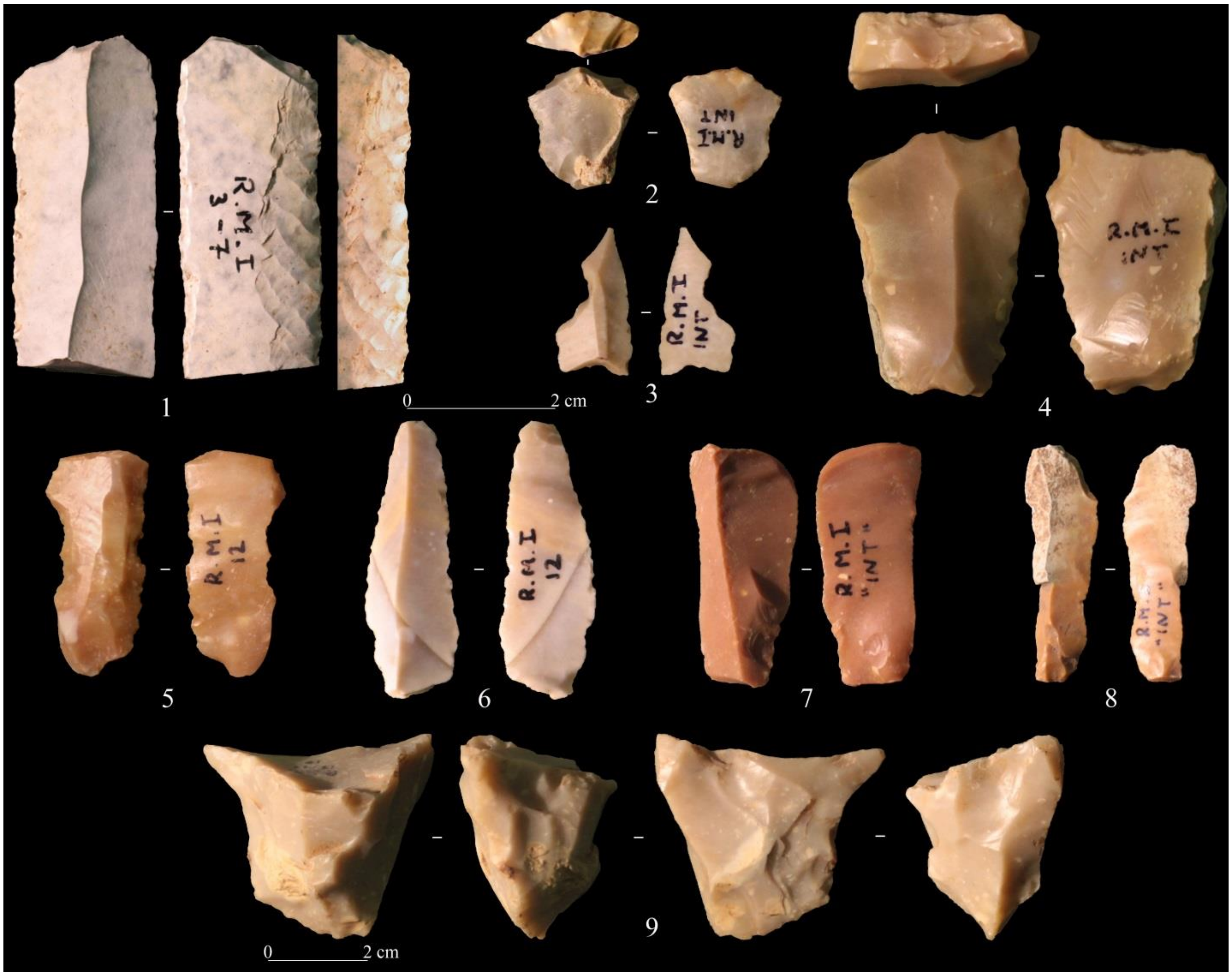

Supplementary Figure S3 – Riparo Mezzena, layer I. Middle Palaeolithic lithic remains. 1: Quina scraper; 2-3: Mousterian points. Scale bar 2 cm. The photos of the lithic assemblage were authorized by the Ministry for Cultural Heritage and Activities - Soprintendenza for Archaeological Heritage of Veneto, and taken by F. Romagnoli; reproduction forbidden.

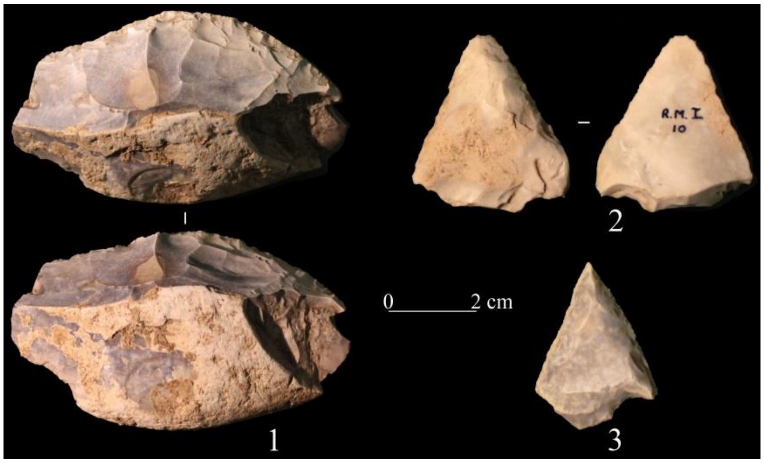

Supplementary Tables:

Supplementary Table S1. Overview of library preparation and sequencing results of the Mezzena samples. C to T substitution frequencies at the terminal positions of sequence alignments are reported in the last four columns.

Library ID	Specimen	Powder used for extraction (mg)	Number of molecules in library (ddPCR)	Number of sequences generated	Number of unique mtDNA sequences	Average number of sequence duplicates	Number of sequences with terminal C→T substitutions	All sequences		Sequences with C→ T at the opposing end	
								**5' C→T (%) [95% CI]**	**3' C → T (%) [95% CI]**	**5' C→T (%) [95% CI]**	**3' C → T (%) [95% CI]**
A9245	IGVR 203334	9.6	3.15E+09	2,532,231	7,903	15.5	181	**5.6** [4.6-6.8]	**8.7** [7.1-10.6]	**31.6** [15.4-54]	**26.1** [12.5-46.5]
R5296	IGVR 63017-4	10.9	4.13E+09	3,301,407	976	1.7	1	**0.4** [0.1-2.3]	**0.8** [0.1-4.1]	**100** [20.7-100]	**100** [20.7-100]
R5297	IGVR 63017-15	15.2	2.73E+09	3,685,080	2,774	2.1	110	**10.4** [8.2-13.1]	**16.2** [12.7-20.5]	**33.3** [18.0-53.3]	**57.1** [32.6-78.6]
R5298	IGVR 63017-2	20.7	2.85E+09	2,502,225	956	1.4	46	**13.0** [9.1-18.2]	**21.2** [14.4-30.0]	**60.0** [23.1-88.2]	**60.0** [23.1-88.2]
R5299	IGVR 63017-12	18.5	5.65E+09	3,143,693	1,767	1.4	8	**1.1** [0.4-2.7]	**1.3** [0.5-3.2]	**0** [0-65.8]	**NA**
R5563	IGVR 63017-5	21.6	1.38E+10	886,577	7,258	51.7	38	**1.4** [0.9-2.0]	**1.3** [0.8-2.2]	**0** [0-65.8]	**0** [0-35.4]
R5564	IGVR 63017-3	20.3	2.73E+09	712,167	5,390	57.1	209	**9.6** [8.1-11.4]	**16.2** [13.4-19.4]	**27.3** [13.2-48.2]	**60.0** [31.3-83.2]
R5565	IGVR 63017-11	18.9	1.42E+09	1,025,260	643	650.8	32	**12.4** [0.8-18.8]	**22.2** [13.7-33.9]	**0** [0-39.0]	**0** [0-56.1]

R5566	IGVR 63017-14	19.4	2.58E+09	764,394	4,254	78.6	382	**23.7** [21.2-26.4]	**33.1** [29.0-37.5]	**32.1** [21.4-45.2]	**40.0** [27.0-54.5]
A9250	ENC	-	8.05E+07	234,989	874	30.1	4	**1.4** [0.5-3.9]	**0.7** [0.1-3.7]	**NA**	**NA**
R5300	ENC	-	7.70E+07	738,674	346	2.5	0	**0** [0-5.3]	**0** [0-6.5]	**NA**	**NA**
R5569	ENC	-	2.37E+07	129,046	293	227.5	2	**3.3** [0.9-11.2]	**0** [0-0.9]	**NA**	**NA**
A9251	LNC	-	4.05E+07	122,375	173	69.9	1	0 [0-0.9]	**2.4** [0.04-12.3]	**NA**	**NA**
R5303	LNC	-	4.28E+07	425,248	48	18.8	2	**66.7** [20.8-93.9]	**0** [0-43.4]	**NA**	**0** [0-79.3]
R5570	LNC	-	1.61E+07	90,070	118	391.8	-	**0** [0-10.2]	**0** [0-19.4]	**NA**	**NA**

ddPCR – digital droplet PCR; ENC – extraction negative control; LNC – library negative control; C – cytosine; T - thymine

Supplementary Table S2. The proportion of sequences in Mezzena samples matching the modern human state or the Neanderthal state.

Library ID	Specimen	All sequences		Sequences with C→ T at the opposing end	
		% Human [# of observations]	% Neanderthal [# of observations]	% Human [# of observations]	% Neanderthal [# of observations]
A9245	IGVR 203334	99.72 [1,062/1,065]	0.28 [3/1,065]	100 [16/16]	0 [0/16]
R5297	IGVR 63017-15	99.44 [356/358]	0.56 [2/358]	100 [7/7]	0 [0/7]
R5564	IGVR 63017-3	99.89 [910/911]	0.11 [1/911]	100 [19/19]	0 [0/19]
R5566	IGVR 63017-14	100 [640/640]	0 [0/640]	100 [45/45]	0 [0/45]
